# Identifying ENSO-related interannual and decadal variability on terrestrial water storage

**DOI:** 10.1038/s41598-021-92729-4

**Published:** 2021-06-30

**Authors:** Se-Hyeon Cheon, Benjamin D. Hamlington, John T. Reager, Hrishikesh A. Chandanpurkar

**Affiliations:** Jet Propulsion Laboratory, California Institute of Technology, Pasadena, CA 91109 USA

**Keywords:** Hydrology, Hydrogeology, Hydrology, Natural hazards

## Abstract

We apply two statistical techniques to satellite measurements to identify a relationship between terrestrial water storage (TWS) and El Niño-Southern Oscillation (ENSO). First, we modified and used the least-squares regression of a previous study using longer records. Second, we applied a cyclostationary empirical orthogonal function analysis (CSEOF). Although the CSEOF technique is distinct from the least-squares regression in that it does not consider proxies, each method produces two modes (decadal and interannual), showing consistency with each technique in spatial pattern and its evolution amplitudes. We also compared the results obtained by the two methods for thirty watersheds, of which five watersheds were compared with previous studies. The combination of the two modes explains the total variance in most watersheds showing the role that interannual and decadal ENSO-related signals in understanding terrestrial water storage variability. The results show that the decadal mode, along with the interannual mode, also plays an important role in describing the local TWS.

## Introduction

Terrestrial water storage (TWS) represents vertically integrated water storage systems over land and includes water stored in tree canopies, surface water, soil, groundwater, snow, and ice^1^. While little is known about the absolute amount of TWS, it directly relates to water availability for human and ecosystem use and is of interest to a range of local to regional scale water resources researchers and managers. Changes in TWS also correspond to water mass movement between land and ocean and have recently emerged as the dominant cause of interannual variability in global mean sea level^2–4^. Many studies indicated that regional imbalances and extreme changes in TWS caused by human activities have been increasing, and this trend is expected to continue in the current century^5–7^. Under these circumstances, it is essential to properly understand the change of TWS and predict the change of TWS based on the understanding in terms of securing stable water resources for human beings and the ecosystem. In particular, the understanding of the natural variability of TWS can provide essential information for estimating human-induced TWS change and also can be used to predict the near future variability of TWS by combining it with predictive information from connected climate modes. Many studies have been carried out to quantify natural fluctuations in TWS and separate these from possible anthropogenic influences^8–12^. One of the primary drivers of the natural variability of TWS is the El Niño-Southern Oscillation (ENSO), and its global and regional influence has been described in many studies^13–18^.

Among studies investigating the ENSO-related TWS signal, few have been conducted on a global scale, and most have relied on comparisons to climate indices tracking the strength of ENSO. Ni et al.^18^, for example, found that interannual TWS changes are strongly correlated with ENSO over much of the globe using multiple data sources, focusing on five river-basins. When it comes to global scale studies, Phillips et al.^9^ (hereafter PH12) estimated the global TWS signal using satellite-measured TWS data over 2003–2010 associated with ENSO by applying a least-squares regression (LSR) leveraging the Multivariate ENSO Index (MEI)^19^. While PH12 suggests a relationship between ENSO and TWS variability in the globe, questions remain regarding the degree to which the MEI represents ENSO and the appropriateness of the underlying technique itself (see Chao and Chung^20^; hereafter CC19). PH12 was conducted on only eight years of data, and with the influence of longer-timescale natural variability during this period, the degree to which ENSO influences can be separated from other variability is challenging to assess^21^.

A non-parametric approach such as Empirical Orthogonal Function (EOF) or Cyclostationary EOF analysis (CSEOF)^22, 23^ has a potential benefit as it does not depend on the predefined climate indices such as MEI and Pacific Decadal Oscillation (PDO)^24, 25^. EOF and CSEOF analysis decompose spatio-temporal data into the statistical modes in the order of each mode’s variance. Various studies have applied CSEOF analysis to decompose climate datasets into independent modes representing natural variabilities^3, 21, 22, 26–28^. The most challenging CSEOF analysis issue is interpreting the decomposed statistical mode, and the previous studies have tried to link the decomposed mode to predefined climate indices.

This study aims to identify and isolate the ENSO-related variability in TWS using the two techniques for satellite measurement. In the first approach, we update and modify the technique of PH12 using the longer data record now available and improved the treatment and use of MEI to respond to the CC19’s concerns. The second approach relies on CSEOF analysis by identifying the ENSO-related statistical modes. After extracting the ENSO-relating TWS variabilities from both techniques, we examine the isolated TWS changes on both global and regional scales.

## Data and methods

### Terrestrial water storage data

While TWS has traditionally been a problematic state variable to measure globally^29^, monthly global observations of TWS change have been made possible by the Gravity Recovery and Climate Experiment (GRACE) mission since 2002^30, 31^. GRACE data have been widely used in hydrology research from regional to global scale on topics such as hydrologic extremes, human water management, changes in surface water, lakes and reservoirs, and groundwater, and the natural and forced variability in storage at multiple time scales (see McCabe et al.^32^ for a summary). The first GRACE satellites ceased their mission in 2017 after roughly 15 years of operations, and GRACE Follow-On (GRACE-FO) has been measuring changes in TWS since May 2018^33^.

In this study, we used RL06M Version 2.0 GRACE MASCON Solution dataset generated by NASA Jet Propulsion Laboratory; in this dataset, a 3° × 3º mascon has one representative value to minimize the observation error^31, 33–35^. This gridded dataset has a one-month temporal and 0.5-degree spatial resolution, and we extracted land from Jan 2003 to Dec 2016 due to the missing period between GRACE and GRACE-FO.

### Least-squares regression

PH12 extracted TWS variability at each grid point of the GRACE data using the MEI and Hilbert-transformed MEI as independent variables in an LSR (Eq. (1)). Ph12 then estimated the amplitudes and phases using regression coefficients to assess the relationship between ENSO and TWS variability at each location around the globe; the amplitudes represent the ENSO’s influence on TWS, and the phase shows the lag between MEI and corresponding TWS changes.

1 $$T\left( {r,t} \right) = Const\left( r \right) + m_{1} \left( r \right) \times MEI\left( t \right) + m_{2} \left( r \right) \times MEI_{H} \left( t \right),$$

where $T\left( {r,t} \right)$ is a space–time ($r$ and $t$) data, $Const\left( r \right)$ is the constant parameter, $m_{1} \left( r \right)$ and $m_{2} \left( r \right)$ are LSR parameters for independent variables, $MEI_{H} \left( t \right)$ is the Hilbert-transformed $MEI\left( t \right)$.

PH12 was conducted on only eight years of data, which is insufficient to capture the influence of longer-timescale natural variability^21^. Also, post-2010, there were two major ENSO events: the 2010–2011 La Niña, which caused the global mean sea level to drop by 7 mm and the 2015–2016 El Niño, one of the biggest in satellite observation era^13^. The inclusion of these extreme events in the current analysis is likely to increase the identified ENSO-TWS relationship’s robustness.

**Figure 1:**
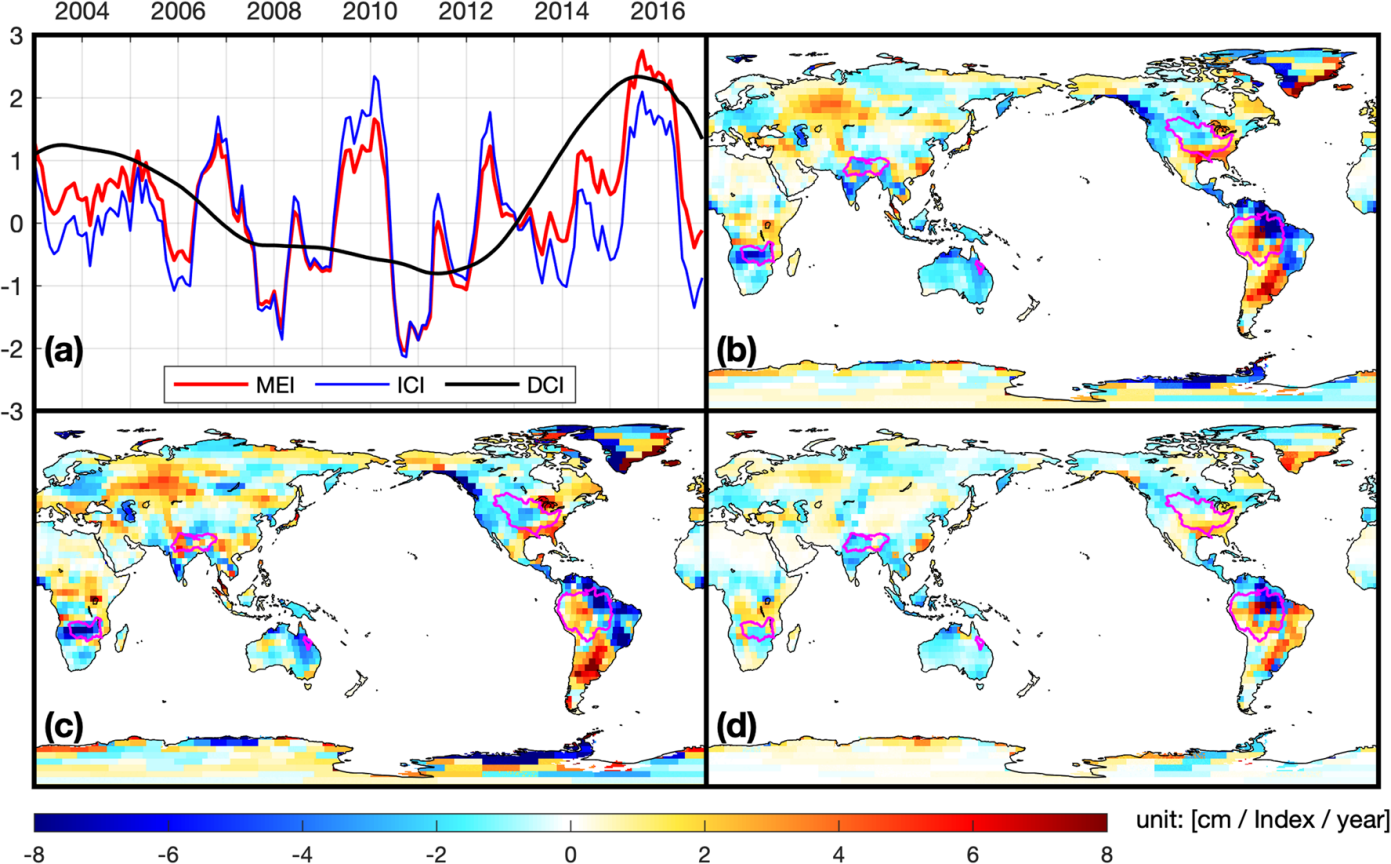
(**a**) ENSO Indices (MEI, DCI, and ICI; normalized by each standard deviation); the spatial map of influence of (**b**) MEI, (**c**) DCI, and (**d**) ICI from the LSR analysis ((**b**) original LSR; (**c,d**) modified LSR); the magenta-colored lines on the maps are five river-basins. The graph and maps were created using MATLAB (version R2020b; https://www.mathworks.com/products/matlab.html).

As a potential remedy to problems arising CC19, we increase the number of independent variables in the regression and separate timescales of variability by decomposing the MEI into decadal variability (or Decadal Climate Index, hereafter DCI) and interannual variability (or Interannual Climate Index, hereafter ICI) following Zhang and Church^36^. We estimated DCI by applying a low-pass filter to the MEI in successive 25 and 37 months moving averages. This is different from Zhang and Church^36^ which used a PDO index to compute the DCI. Here, we are interested in ENSO-related variability and ensuring independent indices, so we used the MEI to compute both the ICI and DCI. After subtracting the DCI from MEI, the remaining signal is the ICI (Fig. 1a). Four independent variables were created by Hilbert transforming both DCI and ICI, and the parameters of each independent variable were calculated through Eq. (2):

2 $$T\left( {r,t} \right) = Const\left( r \right) + d_{1} \left( r \right) \times DCI\left( t \right) + d_{2} \left( r \right) \times DCI_{H} \left( t \right) + i_{1} \left( r \right) \times ICI\left( t \right) + i_{2} \left( r \right) \times ICI_{H} \left( t \right),$$

where $d_{1} \left( r \right)$, $d_{2} \left( r \right)$, $i_{1} \left( r \right)$, and $i_{2} \left( r \right)$ are LSR coefficients, and $DCI_{H} \left( t \right)$ and $ICI_{H} \left( t \right)$ are the Hilbert-transform of $DCI\left( t \right)$ and $ICI\left( t \right)$. Following PH12, we analyzed the ENSO-related changes quantitatively by applying Eq. (3):

3 $$AMP = {\text{~}}\sqrt {A_{1}^{2} + A_{2}^{2} },$$

where $A_{1}$ and $A_{2}$ are LSR coefficients in Eqs. (1) and (2); e.g., $m_{1}$ and $m_{2}$ in Eq. (1).

We removed annual signals and linear trends at each grid location like PH12 before applying the LSR. Then, we estimated the TWS amplitudes of MEI, DCI, and ICI but did not apply the phase analysis of PH12 because of its inaccuracy (CC19).

### Cyclostationary EOF analysis

CSEOF analysis decomposes spatiotemporal data into spatial patterns (or Loading Vector, hereafter LV) and its temporal evolutions (or Principal Component Time-series, hereafter PCT) for each mode. It is similar in structure to the widely used EOF analysis (Eq. (4)), although with the notable difference that the spatial patterns of each mode are periodic, with a specific nested period (Eq. (5)).

4 $$T\left( {r,t} \right) = \mathop \sum \limits_{i} LV_{i} \left( {r,t} \right) \times PCT_{i} \left( t \right),$$

5 $$LV\left( {r,t} \right) = LV\left( {r,t + d} \right),$$

where $T\left( {r,t} \right)$ is the space–time data being decomposed, $LV\left( {r,t} \right)$ is the loading vector, $PCT\left( t \right)$ is the principal component time-series, and $d$ is a nested period.

The nested period’s determination plays a critical role in the CSEOF analysis, especially when targeting specific frequencies, as in this study. Based on the previous studies^21, 37, 38^ about ENSO variability, we chose a 24-month nested period. This 2-year nested period allows for the separation of ENSO’s biennial oscillation (i.e., the transition between warm and cold events) and lower-frequency variability found centered in the Pacific Ocean. The references above provide further justification for the use of this nested period. Before applying CSEOF analysis, we preprocessed the dataset like the LSR analysis.

A brief description of the CSEOF analysis we performed is as follows. First, we performed a harmonic analysis with a 24-frequency-divisions at each grid point of the data^23^. The resulting time-series of each frequency’s coefficient are rearranged into one matrix and performing the principal component analysis (PCA) using the matrix. The PCA outputs were principal component time-series and loading vectors of harmonic components, then using the harmonic components, we recovered the spatial loading vectors by adding up the recovered harmonic signals. Because we used 24 months frequency division, the recovered signals from the coefficients were 24-months repeating feature.

## Results and discussion

### Least-squares regression

We used and modified PH12 to estimate ENSO-related TWS from the GRACE data over 2003–2016. Figure 1b–d show the amplitude maps resulting from these analyses (for more details, see Figs. S1–S3). The amplitude map relating to MEI (Fig. 1b) has relatively large signals in southern Africa, South America, the Antarctic coast, and Greenland. This pattern is generally similar to PH12, but it shows differences in Eastern Asia, South America, Greenland, and northern Australia. This difference may be in part to using six additional years of data compared to PH12, as stated in CC19.

This study further decomposed the MEI into DCI and ICI and applied it to the newly proposed LSR (Eq. (2)). Figure 1c,d show the amplitudes associated with the DCI and ICI, respectively. The ICI’s amplitude map has relatively large values in southern Greenland, South America, and the southeastern United States. The DCI has large amplitudes in other regions, such as southern Africa, South America, North America, and Greenland. Comparing to the MEI map, the DCI and ICI maps appear to be decomposing the result using only the MEI into interannual and decadal components. The similarity between the two maps is not unexpected, given the MEI decomposition in the approach’s structure. For example, significant values in the amplitude using the MEI (see southern Africa, the eastern part of Australia, the southern part of South America, and Central Asia) do not appear in the ICI pattern. These disagreements suggest that larger amplitude areas are mainly represented by the DCI, which is difficult to ascertain using the original LSR from PH12.

While these results do not conclusively tie TWS variability at every location with large amplitudes to ENSO, they demonstrate the climate index-based approach’s potential problems. The range of timescales represented by the MEI can lead to the suggestion that the phase transition from El Niño to La Niña, for example, plays a significant role in the variability at a given location, when there is substantial lower-frequency variability that is driving TWS change at that location.

### CSEOF analysis and comparison with LSR

**Figure 2:**
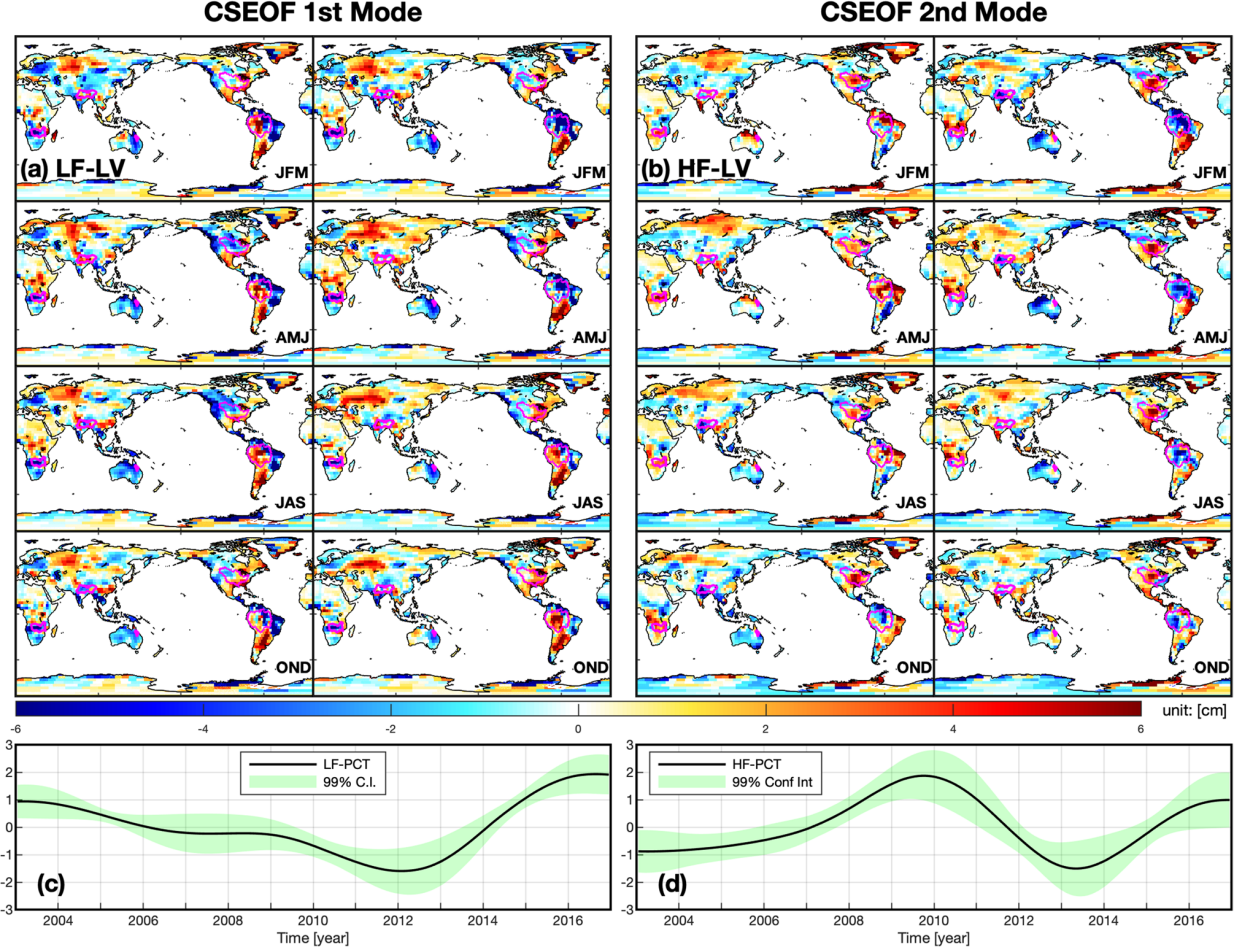
The dominant modes of the CSEOF analysis of GRACE: (**a**) LV of low-frequency mode (3 months averaged), (**b**) LV of high-frequency mode (3 months averaged), (**c**) PCT of low-frequency mode, and (**d**) PCT of high-frequency mode; the magenta-colored lines on the maps are five river-basins; see Figs. S5 and S6 for original LVs. The maps and graphs were created using MATLAB (version R2020b; https://www.mathworks.com/products/matlab.html).

**Figure 3:**
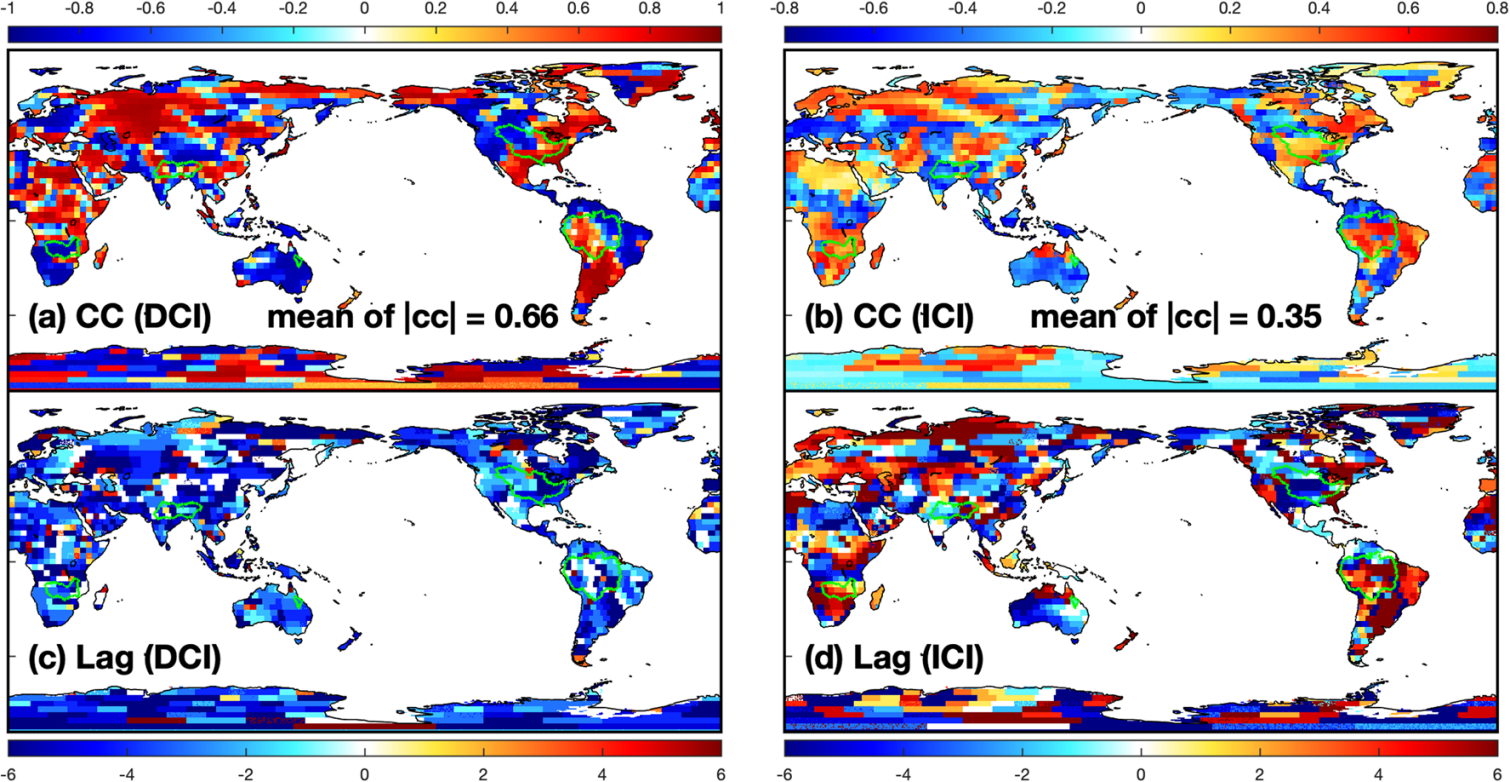
The maximum cross-correlation coefficient and corresponding lag between: (**a,c**) the decadal climate index (DCI) and CSEOF LF-mode at each grid, (**b,d**) the interannual climate index (ICI) and CSEOF HF-mode at each grid. These maps were created using MATLAB (version R2020b; https://www.mathworks.com/products/matlab.html).

Ten CSEOF modes explained 99.9% of GRACE data’s total variance, and the first two dominant modes explained 31% and 21% of the total variance, respectively (for more details, see Figs. S4–S6). Figure 2 shows the two dominant CSEOF modes, and the two modes are highly correlated with ENSO. The maximum cross-correlation coefficient ($\rho$) between a smoothed MEI (6-month moving averaged) and the first and second modes’ spatial-mean time-series (obtained by combining the PCT and spatial-mean of the LV) are − 0.61 and − 0.35, respectively (significant at the 0.05% significance level), and other modes have smaller values than these (the cross-correlation coefficient of each PCT and MEI is given in Fig. S3b). Also, the areas having relatively strong signals in the first and second modes are generally in line with areas known as ENSO-correlated regions^9, 13, 18, 39^ (Fig. 3).

In the first mode (Fig. 2a,c), the spatial patterns change little throughout the 24-month nested period in most areas (but not all the areas), indicating that the dominant physical process described has a temporal evolution of substantially longer than the chosen nested period; the PCT varies only over relatively longer timescales than the second mode. A spectrum analysis using the PCT shows that most of the energy of the PCT is located where the frequency < 0.2 (i.e., return period > 5-years) and more than half of the energy is located where frequency < 0.1 (i.e., return period > 10-years) (see, Fig. S7a). The spectral analysis indicated that the PCT of the first mode has the decadal oscillation. Therefore, from the LV and PCT, we will call this mode the low-frequency (LF) mode and representative of variability on decadal timescales. In the second mode (Fig. 2b,d), the LV shows more frequent changes than the first mode. In many locations, the LV shows a complete cycle of phase transition throughout the 24-month nested period (see the Amazon basin, for example), and the spectral analysis shows that the second mode’s PCT has more energy where the frequency > 0.1 and the largest spectrum value is located in between 0.1 and 0.2 (see Fig. S7b). We conclude the second mode PCT has interannual oscillation with a shorter fluctuation period than the first mode by the spectral analysis. Therefore, we will call this mode the high-frequency (HF) mode representing interannual variability.

To further investigate these modes, we compare the CSEOF modes to the LSR modes. As constructed, the LSR extracts TWS signals corresponding to MEI’s lower and higher-frequency components (DCI and ICI). The LSR subsequently produces two corresponding spatial patterns: one for ICI-mode and DCI-mode. First, we compare DCI-mode and LF-mode. The $\rho$-value between the PCT of the LF-mode and DCI is high as 0.90, and the spatial patterns of the two modes are also very consistent in most areas (Figs. 1c, 2a). We recasted the LF-mode spatiotemporal signals by combining the LF-mode’s LV and PCT to check the cross-correlation between LF-mode and DCI. The maximum $\rho$-value maps of DCI and the recast LF-mode indicates the LF-mode closely correlated to DCI (Fig. 3, Supplemental Fig. S4b). From these results, we conclude that the DCI-mode and LF-mode are very similar, and they represent the same variabilities on TWS.

To compare the ICI-mode and HF-mode, we recasted the HF-mode same as LF-mode. Then, we compute the global spatial-mean time-series of the HF-mode and compare it to the ICI, obtaining $\rho$ of − 0.35. The negative correlation is in line with the fact that ENSO events’ negative phase increases TWS globally and reverses during the positive phase (e.g., Boening et al.^13^). Locations showing substantial variability are similar in both higher-frequency modes, e.g., Australia and Southern Africa. We can see the HF-mode and ICI mode are related to each other, although the similarity is weaker than the lower frequency modes.

### Local ENSO-related TWS

**Figure 4:**
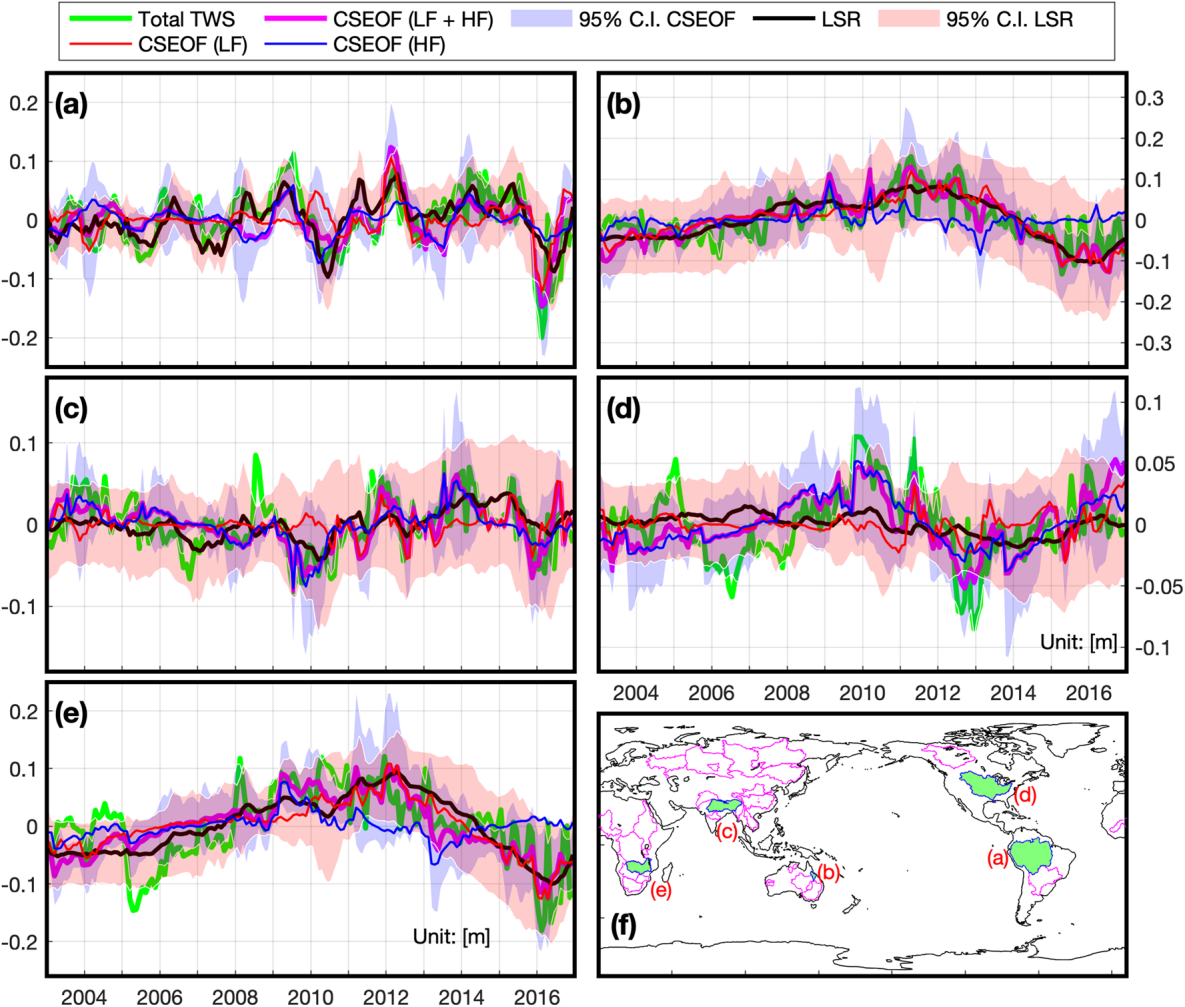
Spatial-mean time-series of regional ENSO-related TWS; (**a**) the Amazon River, (**b**) the Burdekin River, (**c**) the Ganges Brahmaputra River, (**d**) the Mississippi River, (**e**) the Zambezi River, and (**f**) the location of each river-basin (green-filled areas: (**a–e**) and magenta lines: for Table 1); Green: total TWS, Orange: ENSO-related TWS by CSEOF (LF + HF), Black: ENSO-related TWS by LSR, Red: CSEOF LF-mode, Blue: CSEOF HF-mode. The map and graphs were created using MATLAB (version R2020b; https://www.mathworks.com/products/matlab.html).

The ENSO-related TWS of LSR and CSEOF analysis were compared in 30 watersheds to estimate dominant drivers of the regional TWS changes (basins’ information obtained from CEO Water Mandate (http://riverbasins.wateractionhub.org/http://riverbasins.wateractionhub.org/); see Table 1). Among the thirty-basins, we examined five-basins (Amazon, Burdekin, Ganges–Brahmaputra, Mississippi, and Zambezi) in detail. Before the analysis, we made basins’ spatiotemporal datasets: original GRACE data with no annual and linear trend signals, TWS by modified LSR, TWS combining LF and HF-modes, TWS of LF-mode, and TWS of HF-mode. The spatial-mean time-series were estimated in each river-basin using these datasets. The five-basins’ resulting time-series and the percentages of variance explained in the total TWS are shown in Fig. 4 and Table 1.

**Table 1:** Percentage of the variance of total TWS explained for river-basins around the world.

Basins	LSR				CSEOF		
	PH12	Current Study					
	MEI	ICI	DCI	DCI + ICI	HF	LF	HF + LF
(a) Amazon	44.3	34.4	11.2	45.6	20.4	28.7	49.1
Amur	5.3	2.4	11.8	14.2	11.7	14.0	25.7
(b) Burdekin	32.9	7.5	57.8	65.3	10.6	62.9	73.5
Congo	7.7	8.5	1.3	9.8	32.1	9.8	41.9
Eyre lake	17.5	5.8	30.5	36.3	4.9	41.9	56.8
(c) GangesBrahmaputra	10.4	10.4	12.2	22.6	37.1	14.8	51.9
Gascoyne	30.9	24.9	11.1	36.0	38.4	15.7	54.1
Godavari	10.9	18.8	2.6	21.4	23.7	10.1	33.8
Hwang he	5.4	1.8	8.8	10.6	13.4	12.1	25.4
Indus	8.6	2.7	18.2	20.9	8.6	27.8	36.4
Irrawaddy	9.8	7.7	3.8	11.5	11	15.6	26.6
Lena	5.8	3.9	8.7	12.6	9.4	8.6	18
Limpopo	15.9	13.5	4.9	18.5	12.9	11.9	24.8
Mackenzie	21.2	14.8	15.1	29.8	10.0	23.3	33.3
Mekong	15.2	15.4	2.5	17.9	16.3	26.8	43.1
(d) Mississippi	3.2	2	5.1	7.1	40.5	11	51.5
Murchison	37.5	23.9	22.3	46.3	31.3	26.8	58.1
Murray-Darling	19.4	9.9	20.7	30.6	17.7	29.2	46.9
Niger	28.4	17.2	19.8	37	19.8	34	53.8
Nile	3.4	3.3	18.8	22.1	12.1	31.5	43.6
OB	30.1	7.4	54.3	61.8	9.9	60.1	70.1
Okavango	31.5	13.2	45.9	59.1	10.5	50.9	61.4
Orange	19.1	8.8	32.7	41.5	5	42.6	47.6
Parana	28.9	14.6	25.7	40.4	24.0	23.5	47.5
Roper	13.4	11.7	6.2	18.0	29.6	15.8	45.4
Victoria	17	15.3	6.7	22.0	34.6	17.1	51.7
Volga	13.8	6	16.4	22.4	5.7	29.4	35.2
Yangtze	14.3	12	7.9	19.9	10.7	16.6	27.2
Yenisei	2.2	2.2	0.9	3.0	21.1	13.5	34.6
(e) Zambezi	24.9	4.8	51.7	56.5	10.0	45.6	55.6

Previous studies have shown that TWS in the Amazon River is correlated with ENSO^9, 18, 40^. Chen et al.^14^ showed that ENSO is related to extreme TWS changes such as floods and drought in the Amazon river-basin. Figure 4a shows consistency with Chen et al.^14^, showing that 2015–2016 El Niño causes drought, but La Niña (2010–2011) disagrees with the observation. The observed TWS shows the increase in 2011–2012 when the La Niña is weaker than 2010–2011, and it agrees with the previous studies^41, 42^; the LF mode (or PDO) intensifies the increase of TWS. Basin-wide comparisons are complicated because the dynamic spatial variance presented in this region creates difficulties in explaining the regional TWS using an ENSO index. LSR and CSEOF show similar performance by explaining 45.6% and 49.1% of total TWS in this area, and HF and LF-modes describe 20.4% and 28.7% of total TWS. The HF-mode is related to the ICI and explains only 20.4% of TWS. The low-frequency driver plays an essential role in understanding the TWS of the Amazon River basin along with the interannual driver represented by ENSO.

Rainfall and river flow in the northeastern Queensland region of Australia are known to be linked to PDO and ENSO^43, 44^. The Burdekin River is also located in the northeastern Queensland region, and Lough^44^ showed that the river flow was correlated with Nino 3.4 and PDO (correlation coefficients of − 0.29 and − 0.28, respectively). In this study, the LSR and CSEOF describe 65.3% and 73.5% of the TWS of this area, respectively. The LF and HF-modes explain 62.9% and 10.6% of the total TWS, showing that the low-frequency driver is dominant. However, it is consistent with Lough’s results that rainfall and river flow in this area are closely related to PDO, although the connection between TWS and the low-frequency driver is more important here than Lough’s results.

The Ganges–Brahmaputra River basin is negatively correlated with ENSO due to the decrease in TWS when El Niño occurs^17, 45, 46^. In this region, the ENSO-related TWS is not described by ENSO-related indices well because CSEOF explains more of the TWS variance than the LSR, with LSR and CSEOF accounting for 22.6% and 51.9% of TWS, respectively. The HF and LF-modes account for 37.1% and 14.8% of TWS, indicating that ENSO is the primary driver of the TWS change. The regional average of the $\rho$-value of HF-mode is about − 0.42 (Fig. 3). Although ENSO is the primary driver, the LF-mode contributes significantly as well. For example, the TWS changes in Dec-2011 to Jan-2012 and Dec-2015 to Jan-2016 are challenging to explain without the LF-mode. Therefore, the interannual ENSO variability is the dominant driver of regional TWS changes, but the regional TWS can be better explained by also considering the low-frequency driver.

During El Niñ﻿o, Walker Circulation^40^ weakens and causes rainfall in the south-eastern United States^47^. Many studies have been conducted to clarify the relationship between the water resources and ENSO in the Mississippi River basin^48–53^. Munoz and Dee^52^ explained the increase in the probability of flooding after El Niño as the increase in rainfall in the southern Mississippi River basin due to El Niño and the resulting increase in soil moisture saturation. Thus, El Niño positively correlates with TWS in the southern part of the Mississippi River basin (Fig. 3). However, it is challenging to explain the TWS of this basin using ENSO only because positive and negative relations exist simultaneously (Fig. 3). In this area, LSR only accounts for 7.1% of TWS, whereas CSEOF can describe 51.5% of the region’s TWS. The result only indicates that the proxies could not pick up the ENSO-related TWS, not indicates that there is no ENSO-related TWS because the extracted CSEOF’s ENSO-related TWS describes more than 51% of the total variance of regional TWS. HF and LF-modes account for 40.5% and 11% of the regional TWS, showing the HF-mode is dominant. The TWS of this region is linked to ENSO, but its impacts are not easy to separate from other TWS variability. It is challenging to describe the regional TWS using ENSO-related indices, but CSEOF’s HF and LF-modes can better describe the regional TWS variability.

Gaughan et al.^27^ showed that El Niño (La Niña) was associated with an increase (decrease) in rainfall intensity in the Zambezi River basin. However, in this study, the HF and LF-modes account for 10% and 45.6% of the local TWS. The LF-mode explains far more variance than the HF-mode (Fig. 4e, Table 1). The LSR and CSEOF account for 56.5% and 55.6% of the regional TWS, indicating that DCI and ICI are generally able to explain interannual to decadal variability in TWS in the region. Although we could not find strong evidence that TWS in this area is driven by ENSO variability, we did find the description of TWS is improved by including a separate representation of lower-frequency variability (either DCI or LF-mode).

## Summary

To assess ENSO influence on TWS, we conducted LSR analysis after dividing MEI into a decadal index (DCI) and an interannual index (ICI) using the GRACE dataset over 2003–2016. Through the better separation of timescales, some of the issues raised in CC19’ are addressed, and the resulting spatial patterns associated with ICI and DCI are obtained. This analysis shows that the amplitude map using only the MEI in PH12 is separable into two distinct timescales. While this separation may not be surprising, both timescales are represented in the MEI, highlighting the importance of decadal variability in TWS at many locations.

We also performed CSEOF analysis as a proxy-independent scheme, which separates spatiotemporal data into a modal description. It is difficult to assign a particular statistical mode-specific physical meaning, but the two dominant CSEOF-modes correspond with the LSR’s results. The first CSEOF-mode connects closely to DCI-mode, with similar spatial variability and associated time-series. The second CSEOF-mode has an apparent 2-year periodicity and a similar spatial pattern of ICI-mode.

To consider the regional impacts of the lower and higher-frequency modes, we evaluated the results of LSR and CSEOF in thirty watersheds, and five of them were selected for more intensive analysis. We confirmed the roles of the interannual and decadal variabilities of TWS in each region, and through this, we identified the ENSO impact on each region’s TWS. Both interannual and decadal variabilities had essential roles in describing the regional TWS, and their roles differed in each area. Both methods showed consistent results with previous studies in most regions and accounted for the local TWS at similar levels in the Amazon, Burdekin River, and Zambezi River basins. However, in the Ganges–Brahmaputra River and the Mississippi River, the CSEOF analysis described the total TWS far more than the LSR; actually, most basins CSEOF better described the total TWS than LSR. From this, we found the CSEOF could provide the ENSO-related TWS signals even the ENSO-related climate index cannot describe the regional ENSO-related TWS signals.

In conclusion, by modifying PH12’s LSR and applying a new technique (CSEOFs), we have taken steps to isolate the ENSO-related TWS signals on a global scale. However, through the LSR analysis, potential problems have been identified with the approaches relying on a climate index that imposes a particular structure on the extracted variability. In this respect, statistical decompositions such as CSEOF analysis are potentially advantageous in isolating and understanding the natural variability in TWS. This advantage will become more evident as more observations are accumulated in the future. Also, the estimation of ENSO’s impact on thirty watersheds showed that the decadal and interannual changes’ influences were different in each watershed. We expect the two methods shown in this study can increase water resource management capability by combining ENSO’s short-term prediction scheme. Finally, we propose that more investigations (about the physical interpretation of regional ENSO-related TWS and its relationship with other data such as precipitation, runoff, and evaporation) are necessary shortly.

## Supplementary Information

Supplementary Information.

## Data Availability

The JPL GRACE and GRACE-FO Mascon Ocean, Ice, and Hydrology Equivalent Water Height Coastal Resolution Improvement (CRI) Filtered Release 06 Version 2 can be obtained from NASA PO.DAAC, CA, USA, at https://doi.org/10.5067/TEMSC-3JC62. The MEI index can be obtained from NOAA Physical Sciences Laboratory (PSL), Boulder, CO, at https://psl.noaa.gov/enso/mei.old/mei.html.
