# Supplementary material for: Identifying ENSO-related interannual and decadal variability on terrestrial water storage
Source: Sci Rep. 2021 Jun 30;11:13595. doi: 10.1038/s41598-021-92729-4 (PMC8245421; doi:10.1038/s41598-021-92729-4)
Supplement: Supplementary file 1 — Supplementary Information. [file 41598_2021_92729_MOESM1_ESM.pdf]

SUPPLEMENTARY INFORMATION: FIGURES

Identifying ENSO-related interannual and decadal variability on terrestrial water storage

Se-Hyeon Cheon[1,*], Benjamin D. Hamlington[1], John T. Reager[1], and Hrishikesh A. Chandanpurkar[1]

[1]Jet Propulsion Laboratory, California Institute of Technology, Pasadena, CA 91109, USA.
[*]se-hyeon.cheon@jpl.nasa.gov

Introduction

We provide seven figures which can help the readers to have a clear understanding of the current paper.

Figure S1 is the coefficient maps of current study's least squared regression (LSR).

Figure S2 is the standard deviation of Figure S1's coefficient maps.

Figure S3 is the LSR's resulting correlation coefficient map with the target (TWS). The square of this map becomes the R-square map.

Figure S4 is the summary of CSEOF analysis.

Figure S5 and 6 are the loading vectors' spatial patterns of LF-mode and HF mode of the GRACE's CSEOF analysis over 2003-2016. To increase the readability, we provide the seasonal mean of the initial results in the paper. To give the readers a chance to see the original spatial patterns of CSEOF loading vectors, we provide these supporting figures.

Figure S7 is the spectral analysis of CSEOF first and second modes' principal component time series (PCT).

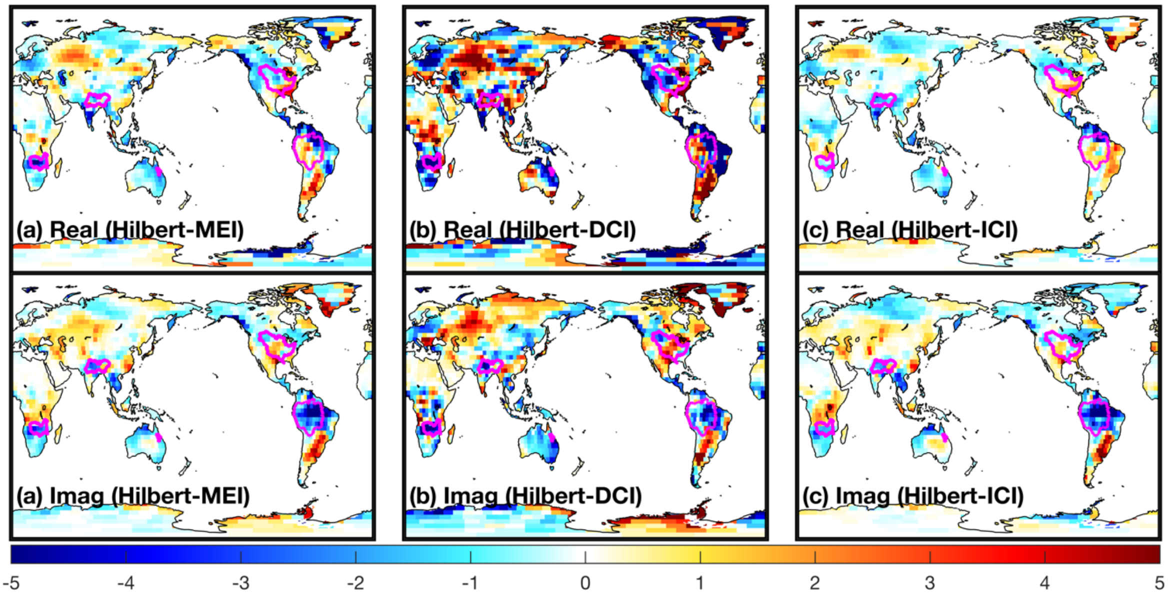

Figure S1. The resulting coefficients of LSR: (a) Hilbert transformed (Ph12), (b) Hilbert transformed DCI, and (C) Hilbert transformed ICI; the magenta-colored lines on the maps are five river-basins. These maps were created using MATLAB (version R2020b; https://www.mathworks.com/products/matlab.html).

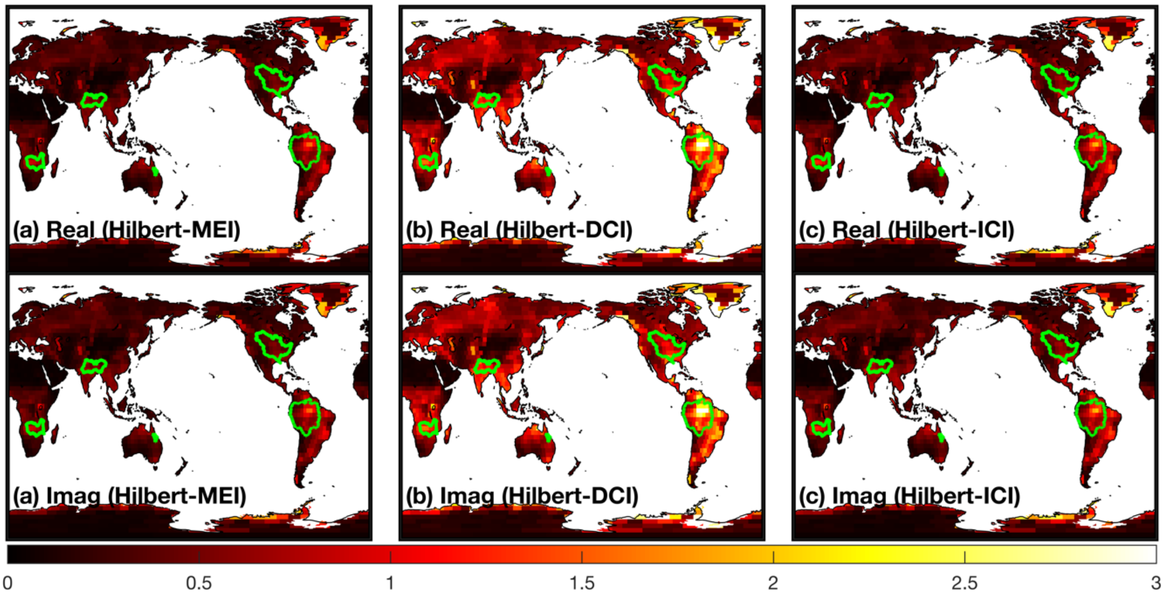

Figure S2. The standard deviation of resulting coefficients of LSR: (a) Hilbert transformed (Ph12), (b) Hilbert transformed DCI, and (C) Hilbert transformed ICI; the green-colored lines on the maps are five river-basins. These maps were created using MATLAB (version R2020b; https://www.mathworks.com/products/matlab.html).

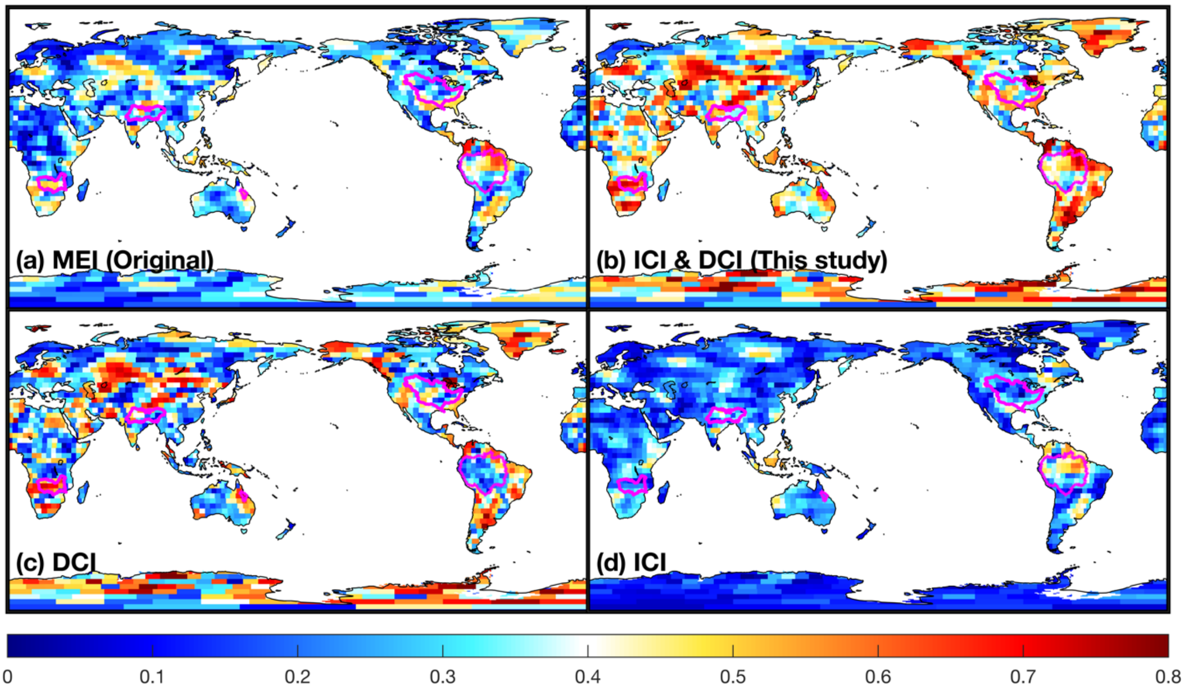

Figure S3. The correlation coefficient between TWS and LSR results: (a) MEI (Ph12), (b) combining ICI and DIC, (c) DCI only, and (d) ICI only; Note: R^2-values of LSR can be obtained by squaring these maps values. These maps were created using MATLAB (version R2020b; https://www.mathworks.com/products/matlab.html).

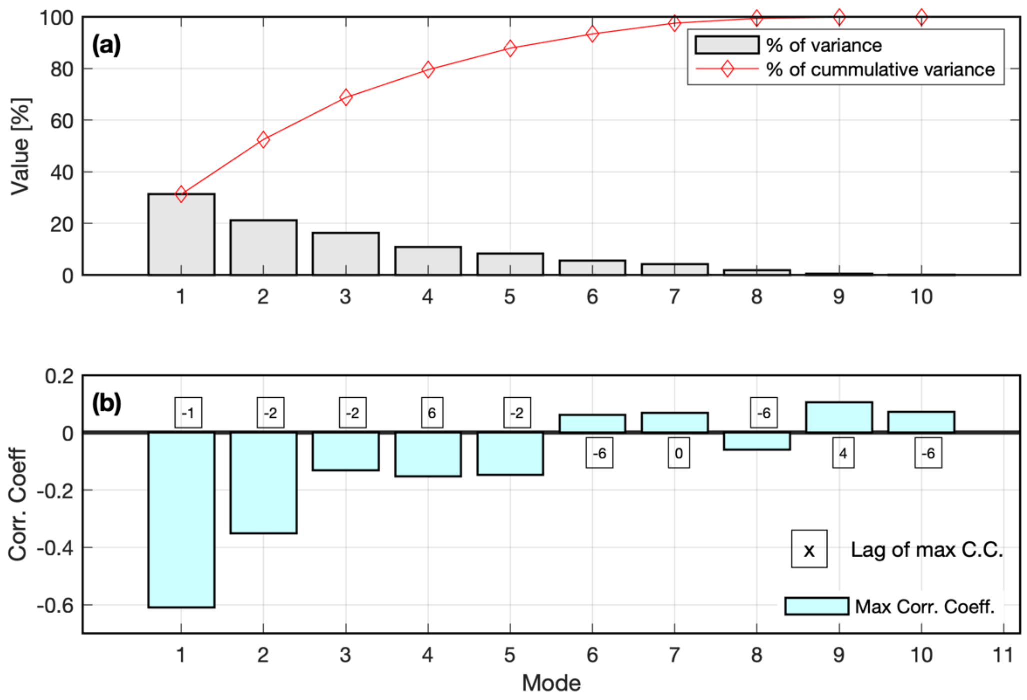

Figure S4. (a) Each CSEOF mode's percent of total variance of GRACE data; (b) cross-correlation between each CSEOF mode's spatial mean time-series and MEI.; for (b) we recast each mode's spatiotemporal data by combining LV and PCT of each mode. This figure was created using MATLAB (version R2020b; https://www.mathworks.com/products/matlab.html).

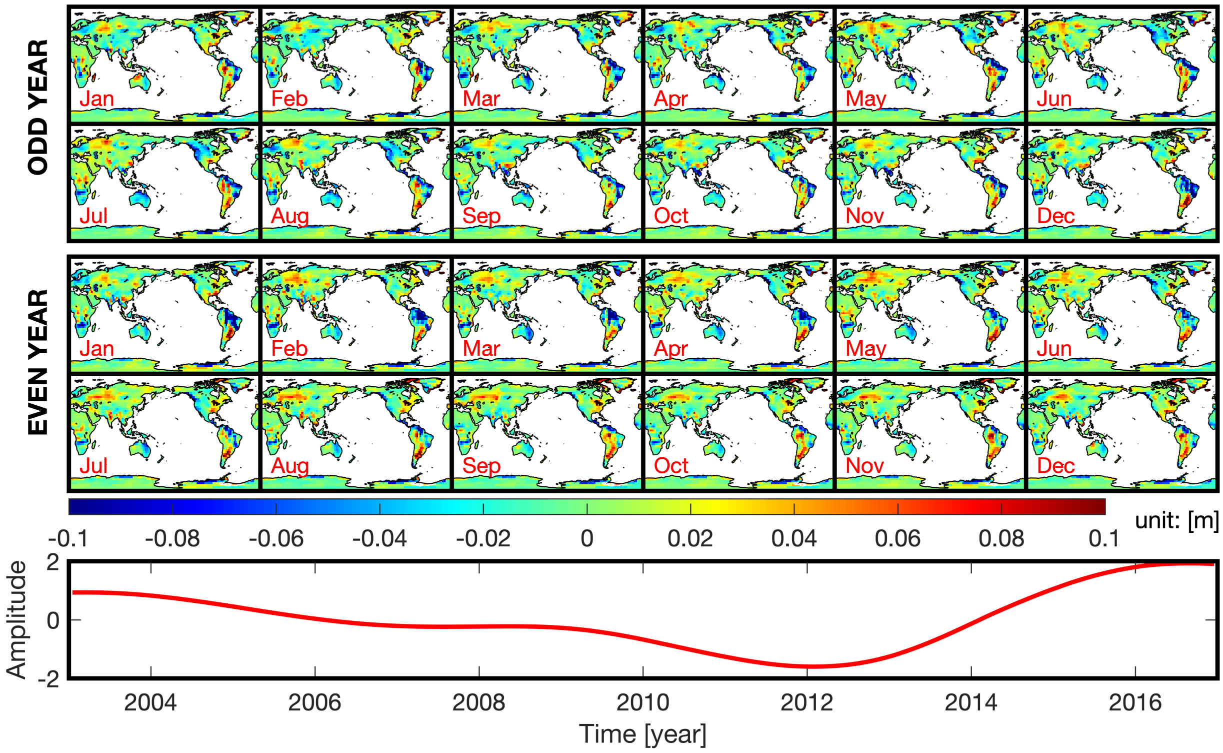

Figure S5. The LV and PCT of the LF-mode of the CSEOF analysis of GRACE. The maps and graph were created using MATLAB (version R2020b; https://www.mathworks.com/products/matlab.html).

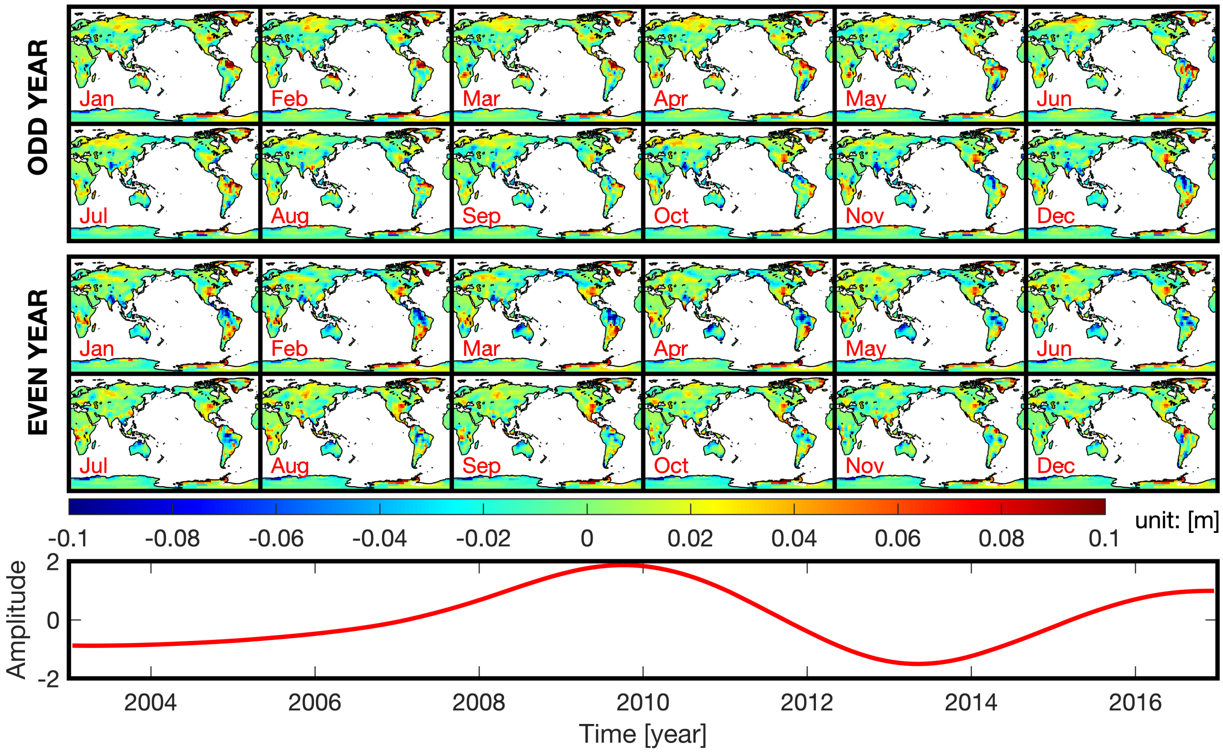

Figure S6. The LV and PCT of the HF-mode of the CSEOF analysis of GRACE. The maps and graph were created using MATLAB (version R2020b; https://www.mathworks.com/products/matlab.html).

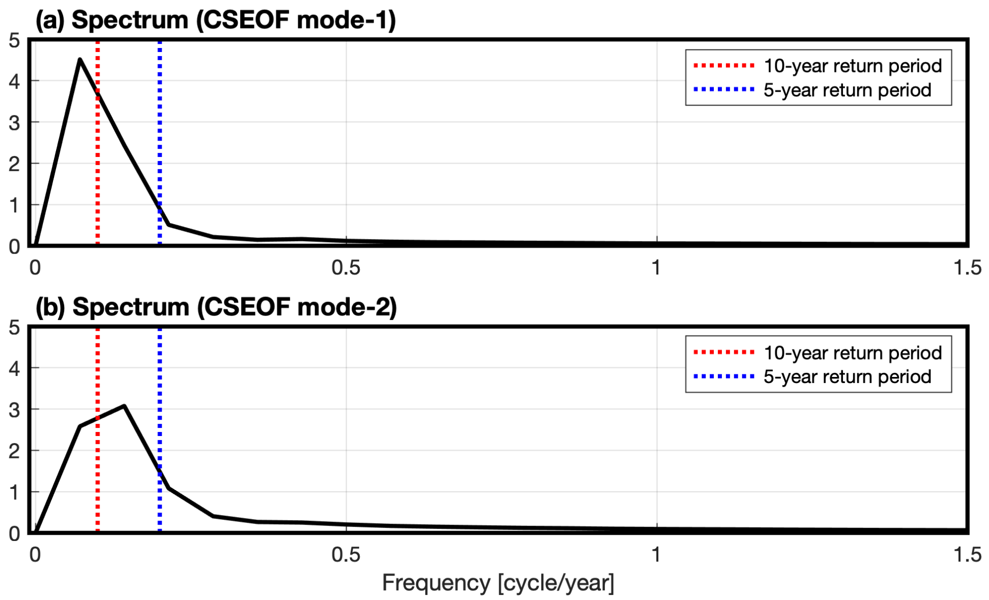

Figure S7. Spectral analysis of CSEOF mode's PCT: (a) LF mode's (the first mode), (b) HF mode's (the second mode). This figure was created using MATLAB (version R2020b; https://www.mathworks.com/products/matlab.html).
